# Association Between Plasma Amyloid‐Beta 42 Ratio and Postoperative Delirium in Elderly Patients Undergoing Major Abdominal Surgery: Secondary Analysis of a Randomized Controlled Trial

**DOI:** 10.1002/brb3.70501

**Published:** 2025-04-21

**Authors:** Qianqian Fan, Yonghui Wang, Zhihong Lu, Lini Wang, Xue Yang, Ziyu Zheng, Hailong Dong, Lize Xiong, Chong Lei

**Affiliations:** ^1^ Department of Anesthesiology and Perioperative Medicine Xijing Hospital Fourth Military Medical University Xi'an China; ^2^ Department of Anesthesiology and Translational Research Institute of Brain and Brain‐Like Intelligence Shanghai Fourth People's Hospital Affiliated to Tongji University School of Medicine Shanghai China

**Keywords:** amyloid‐beta 42, delirium, elderly, abdominal surgery

## Abstract

**Introduction:**

Cerebrospinal fluid Aβ42 has been proposed as a potential indicator for cerebral β‐amyloidosis and may be involved in the pathophysiology of delirium. Whether perioperative plasma Aβ42 alternation is associated with postoperative delirium risk among elderly patients remains unknown.

**Methods:**

This was a secondary analysis of a randomized controlled trial evaluating the effects of acupuncture (intervention) compared to standard care (control) on the incidence of delirium in patients undergoing major abdominal surgery. Participants with blood samples collected were included in this cohort study. The exposure variable was the Aβ42 ratio, calculated with the plasma Aβ42 level immediately after surgery divided by the preoperative plasma Aβ42 level. The primary endpoint was the occurrence of delirium within the first 7 days following surgery or until hospital discharge, whichever happened first, evaluated using either the Confusion Assessment Method or the Confusion Assessment Method‐intensive care unit for intubated patients. Delirium severity was a secondary outcome assessed by the Memorial Delirium Assessment Scale. The logistic regression models and a restricted cubic spline were performed to examine the association between the Aβ42 ratio and delirium incidence, with receiver operating characteristic curve (ROC) analysis for diagnostic power. The mediation effects of the matrix metalloproteinase‐9 ratio were further explored by causal mediation analysis. The linear regression and generalized linear mixed models assessed the association between the Aβ42 ratio and delirium severity.

**Results:**

A total of 195 patients with blood samples collected were included in the final analysis. Among them, the mean age was 70.2 ± 4.2 years; 134 were female (68.7%), and 26 (13.3%) patients experienced postoperative delirium. The plasma Aβ42 ratio was positively correlated with an increased delirium risk (adjusted odds ratio 3.21, 95% confidence interval 1.71–6.05, *p < *0.001) and delirium severity, as measured by the highest postoperative Memorial Delirium Assessment Scale score (adjusted *β* coefficient 3.04, 95% confidence interval 0.9–5.18, *p *= 0.006) in the fully adjusted multivariable analysis models. The restricted cubic spline indicated a linear relationship between the plasma Aβ42 ratio and delirium incidence (*p *= 0.202). The ROC showed that the area under the curve for the Aβ42 ratio to predict delirium risk was 0.698 (95% CI, 0.582–0.814), with the optimal cut‐off point of 0.137. Mediation analyses showed that the Aβ42 ratio does not mediate postoperative delirium through the matrix metalloproteinase‐9 ratio (proportion: 1.3%).

**Conclusions:**

This cohort study showed that a higher Aβ42 ratio was associated with an increased delirium risk and severity, and the association was linear. The plasma Aβ42 ratio might be a mini‐invasive biomarker to identify postoperative delirium.

## Graphical abstract

In a secondary analysis of a randomized controlled trial, we investigated the plasma Aβ42 ratio's association with delirium risk and severity in elderly patients undergoing abdominal surgery. The results showed that a higher Aβ42 ratio was associated with an increased delirium risk and severity, and the association was linear.

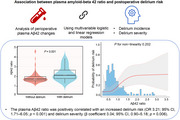

## 1 Introduction

Delirium is a clinical symptom with characteristics of acute onset, mental status, severity fluctuation, inattention, disrupted thinking patterns, and alterations in consciousness and cognition (Marcantonio 2017). Postoperative delirium is one of the most common complications and occurs in approximately 15%–50% of elderly surgery patients (Marcantonio 2017). Postoperative delirium is associated with increased risks of long‐term cognitive dysfunction, prolonged hospital stays, higher healthcare expenditures, as well as increased mobility and mortality (Inouye 2006; Wyrobek et al. 2017). The pathophysiologic mechanisms underlying postoperative delirium remain poorly understood. Neurological injury, disturbances in neurotransmitter pathways, blood‐brain barrier (BBB) breakdown, and systemic inflammation may all play a role (Casey et al. 2020; Lopez et al. 2020; Nadelson et al. 2014; Taylor et al. 2022). Evidence has shown that patients with cognitive impairment or dementia have a higher risk of developing delirium compared to the general population, and delirium is independently associated with the progression of cognitive decline and dementia (Fong and Inouye 2022). Thus, delirium may share common pathological mechanisms with dementia and cognitive disorders such as Alzheimer's disease (Fong and Inouye 2022).

Amyloid‐beta (Aβ) is a peptide formed through the proteolytic cleavage of the amyloid precursor protein, and the accumulation of Aβ in the brain is regarded as a key pathological hallmark of Alzheimer's disease (Carbone et al. 2021). Extracellular deposition of brain Aβ spreads tau tangles, causes neuronal injury, and leads to progressive cognitive decline (Scheltens et al. 2016). Aβ40 and Aβ42 are the main components of extracellular senile plaques in neurodegenerative diseases, while Aβ42 is more toxic and is more prone to aggregate than Aβ40 (Qiu et al. 2015; Scheltens et al. 2016). Cerebrospinal fluid (CSF) Aβ42 has been reported to be a potential indicator of cerebral β‐amyloidosis (Jack et al. 2010). A recent meta‐analysis indicates preoperative CSF Aβ42 levels are negatively related to postoperative delirium risk (Geng et al. 2024). However, the invasive CSF sampling procedure hampers the clinical application of CSF Aβ42 as an indicator of postoperative delirium.

It has been reported that there is a dynamic balance in Aβ levels between the brain and plasma, and changes in plasma Aβ concentrations lead to an alternation of brain Aβ deposition (Bu et al. 2018; Imbimbo et al. 2020; Shi et al. 2024). In animal studies, Bu et al. (2018) documented that blood‐derived human Aβ can enter the brain and cause degenerative changes and amyloid angiopathy. Surgical trauma may disrupt BBB integrity (Taylor et al. 2022), potentially facilitating bidirectional Aβ flux between central and peripheral compartments. Recently, the Food and Drug Administration has authorized lecanemab, an anti‐Aβ monoclonal antibody, as a treatment for early symptomatic Alzheimer's disease, based on its ability to enhance the clearance of Aβ from both plasma and the central nervous system, leading to a declined Aβ burden (Shi et al. 2024). A preliminary study also suggested that high plasma Aβ42 concentrations were linked to later cognitive deterioration in individuals with mild cognitive impairment (Chen et al. 2019). Therefore, plasma Aβ42 level may be a surrogate of CSF Aβ42 and can be used as an indicator for predicting postoperative delirium. Currently, limited research has explored the association between plasma Aβ42 and delirium risk, and the conclusions of these studies have been inconsistent (Geng et al. 2024; Payne et al. 2023; van den Boogaard et al. 2011). It was demonstrated that a single static measurement of plasma Aβ42 is insufficient for accurately predicting cognitive dysfunction (Olsson et al. 2016) or delirium (van den Boogaard et al. 2011), whereas dynamic Aβ42 changes may help predict disease progression (Seppälä et al. 2010). Nonetheless, the perioperative alternation in plasma Aβ42 and the correlation between these changes and postoperative delirium among elderly patients are still unclear and need further investigation (Geng et al. 2024).

In the current study, we conducted a secondary analysis of a randomized controlled trial to investigate the relationship between the plasma Aβ42 ratio (defined as the plasma Aβ42 level immediately after surgery divided by the preoperative plasma Aβ42 level) and the occurrence as well as the severity of postoperative delirium. Our hypothesis was that an elevated plasma Aβ42 ratio would be independently associated with increased delirium risk in elderly patients after major abdominal surgery.

## 2 Materials and Methods

### 2.1 Study Overview and Participants

The data used for the final analysis were sourced from a previous randomized controlled trial conducted at Xijing Hospital in Xi'an, China, from April 17, 2019 to April 10, 2020 (Fan et al. 2022). The parent study (including blood sample collection) has been registered at ClinicalTrials.gov (NCT03726073) and obtained approval from the Institutional Review Board of Xijing Hospital (No. KY20182080‐F‐1). Written informed consent was obtained from all patients. The main objective of the parent study was to evaluate the impact of perioperative acupuncture therapy, with patients randomized into two arms: the acupuncture intervention arm (105 patients) and the standard care control arm (105 subjects). The inclusion criteria were patients older than 65 years, American Society of Anesthesiologists physical status class ≤ III, Mini‐Mental State Examination (MMSE) score higher than 20, and scheduled for elective major abdominal surgery. The exclusion criteria were patients with literacy deficiencies, severe visual or auditory impairments, a history of brain injury or neurosurgery, mental illness, alcohol or drug abuse, contraindications to transcutaneous electrical acupoint stimulation or auricular acupressure, or prior acupuncture treatment. All patients with available blood samples were included in this cohort. This manuscript adheres to the Strengthening the Reporting of Observational Studies in Epidemiology guidelines (von Elm et al. 2014).

### 2.2 Sample Collection and Processing

The blood samples were obtained 30 min before anesthesia induction and immediately after surgery. Immediate postoperative sampling was chosen as this early phase may represent a window for detecting acute pathophysiological responses triggered by surgical trauma, facilitating the exploration of mechanisms linking Aβ42 dynamics to subsequent delirium pathogenesis (Taylor et al. 2022). Samples were collected into tubes containing ethylenediaminetetraacetic acid and processed within 4 h of collection. Samples were centrifuged at 3000 × *g* for 10 min at room temperature, and supernatant plasma was collected and stored in enzyme‐free Eppendorf tubes, which were placed in a −80°C freezer until the assay was performed.

The concentrations of plasma Aβ42, interleukin (IL)‐6, IL‐10, nerve growth factor (NGF), tumor necrosis factor α (TNFα), aquaporin 4 (AQP4), tau, and matrix metalloproteinase‐9 (MMP9) were measured by human enzyme‐linked immunosorbent assay (ELISA) kits (Aβ42 [catalog number: F00133], IL‐6 [catalog number: F01310], IL‐10 [catalog number: F01360], TNFα [catalog number: F02810], MMP9 [catalog number: F01900], AQP4 [catalog number: F02510], Tau [catalog number: F02914], NGF [catalog number: F02040], S100β [catalog number: F02500], Tau [catalog number: F02914], Shanghai Xitang Biotechnology Co., LTD, Shanghai, China). All ELISA measurements were conducted by experienced technicians blinded to the research question of interest and the related clinical information. All antibodies and plates were sourced from the same lot to eliminate the variability among batches. The intra‐batch and inter‐batch coefficients of variation for Aβ42 were < 12%, and for IL‐6, IL‐10, MMP9, AQP4, NGF, TNFα, S100β, and Tau were < 10%.

The exposure variable was the plasma Aβ42 ratio, calculated as the plasma Aβ42 level immediately after surgery divided by the preoperative plasma Aβ42 level. Similarly, the other cytokine ratios were derived by dividing the cytokine levels measured postoperatively by those measured preoperatively.

### 2.3 Delirium Assessment

The primary endpoint was the occurrence of delirium within the initial postoperative 7 days or until the patients' hospital discharge, depending on which occurred first. Delirium was evaluated once daily, approximately at 6:00 p.m. The diagnosis of delirium and its subtype were determined using either the Confusion Assessment Method (CAM) or the CAM for the intensive care unit, specifically for intubated patients. The Richmond Agitation Sedation Scale, patient family feedback, and a thorough examination of medical records further supported this assessment. Details have been described in the parent paper (Fan et al. 2022). Delirium severity was considered the secondary outcome, assessed by the Memorial Delirium Assessment Scale (MDAS). MDAS scores were evaluated for all patients, regardless of whether they met delirium diagnosis.

### 2.4 Clinical Covariates

The clinical data collected included demographics such as gender, age, body mass index, and duration of education. Additionally, the baseline MMSE score and Charlson comorbidity index were recorded. Other data included the American Society of Anesthesiologists class, surgery type, surgery length, anesthesia length, intraoperative consumption of sufentanil and remifentanil, amount of blood loss during surgery, and highest MDAS score.

### 2.5 Statistical Analysis

Given that this study was a secondary analysis of a parent randomized controlled trial, the sample size was constrained by the original trial's design. However, to evaluate the feasibility of detecting associations in this cohort, we performed a post hoc power analysis. Based on the parent study, where the overall incidence of postoperative delirium in this cohort was 12.9% (27/210) (Fan et al. 2022). We hypothesized that patients with a higher Aβ42 ratio (categorized by the median value) might exhibit an elevated delirium risk. Assuming a relative risk of 2.5 for the incidence of the primary outcome (postoperative delirium) between patients with higher and lower Aβ42 ratio groups, a sample size of 195 patients would provide about 90% power at a two‐sided alpha level of 0.05.

For clinical variables, continuous data were presented as mean with standard deviation for variables exhibiting normal distribution, whereas the median with 25th and 75th percentiles was utilized for skewed data. Categorical variables were described in terms of frequencies and proportions. Clinical characteristics were compared using Student's *t*‐tests, Wilcoxon rank sum tests, and chi‐square tests, depending on the nature of the data and the appropriateness of each test.

Univariable and multivariable binary logistic regression models were applied to assess the association between the Aβ42 ratio and delirium risk. The multivariable logistic regression models included covariables that met one or more of the following criteria: (1) confounders previously identified in the literature, (2) covariates with a significance level of *p *< 0.05 in univariable analyses, and (3) covariates that, when included or excluded from the model, result in a change in the effect size of the exposure by more than 10%. Given that postoperative delirium is often regarded as the brain's response to inflammation and BBB disruption, we hypothesized that neuronal injury markers, such as Aβ42, would correlate to some extent with inflammation and BBB disruption markers. We conducted the Spearman correlation analysis to evaluate the association between the Aβ42 ratio and other cytokine ratios. Cytokines significantly correlated with the Aβ42 ratio and survived Bonferroni corrections for multiple comparisons (*p* < 0.006, calculated as 0.05 divided by eight comparisons) and were considered potential covariates in the multivariable regression models.

The receiver operating characteristic (ROC) curve was constructed to evaluate the predictive performance of the Aβ42 ratio for postoperative delirium risk. The area under the curve (AUC) was calculated, and the following metrics were derived from the confusion matrix results: accuracy (defined as the number of correct predictions divided by the total number of predictions), sensitivity, specificity, positive predictive value (PPV), and negative predictive value (NPV). We adjusted the optimism/overfitting in measures of AUC and confusion matrix results using Bootstrap with 1000 resamples. Youden's index was applied to identify the optimal cut‐off value for the Aβ42 ratio by maximizing the sum of sensitivity and specificity. We also tested whether MMP9 mediated the association between the Aβ42 ratio and delirium risk, employing bootstrap resampling (1000 iterations) to estimate direct/indirect effects and a 95% confidence interval (CI).

Linear regression models were employed to assess the association between the Aβ42 ratio and delirium severity, using peak MDAS scores. Aβ42 ratios were log‐transformed in the linear regression analysis due to the violation of the residual normality assumption. Additionally, a generalized linear mixed effects model was applied to investigate the association between the Aβ42 ratio and MDAS scores over time, with the Aβ42 ratio included as the fixed effect and time and participants as the randomized effects. The model covariates were unadjusted.

Subgroup analyses were conducted using logistic regression models to explore the relationship between the Aβ42 ratio and delirium risk across different subgroups varied by age (≤ 70 vs. > 70 years), the randomization group (control vs. intervention), and duration of education (≤ median vs. > median). The statistical significance of interaction effects was assessed in each subgroup.

Several prespecified sensitivity analyses were performed to assess the primary analysis's robustness. First, we employed restricted cubic splines with four knots (located at the 5th, 35th, 65th, and 95th percentiles of the Aβ42 ratio) to flexibly model the relationship between the Aβ42 ratio and delirium incidence. We evaluated models with three to five knots but ultimately chose four knots, as this minimized the Akaike information criterion. The spline models were fully adjusted. Second, to investigate the odds ratio (OR) of delirium risk in different ranges of the Aβ42 ratio, we categorized the Aβ42 ratio into quartiles and included these quartiles as categorical variables in the models. We designated the first quartile of the Aβ42 ratio as the reference group and assigned a median value to each category to do the linear trend tests. Third, changes in plasma Aβ42 levels (calculated by subtracting preoperative plasma Aβ42 level from plasma Aβ42 level immediately after surgery) and log‐transformed Aβ42 ratio were used as alternative approaches to account for the correlation. Fourth, we examined the potential impact of unmeasured confounders by calculating the *E* value to assess the robustness of this relationship between delirium risk and the Aβ42 ratio. The *E* value serves as a metric to quantify the strength of an unmeasured confounder capable of reversing the observed linkage between the Aβ42 ratio and postoperative delirium risk (Haneuse et al. 2019).

Participants with > 10% missingness in covariates were excluded during the final analysis. If less than 10% of covariate data were missing, missing values were estimated using informative or mean imputation, as appropriate. For all analyses, a two‐tailed *p *< 0.05 was considered statistically significant. The analyses were performed using SPSS statistical software version 24.0 (IBM Corporation, Armonk, NY, USA) and the Free Statistics analysis platform (Version 1.9, FreeClinical Medical Technology Co. Ltd., Beijing, China).

## 3 Results

### 3.1 Clinical Characteristics of the Study Population

**FIGURE 1:**
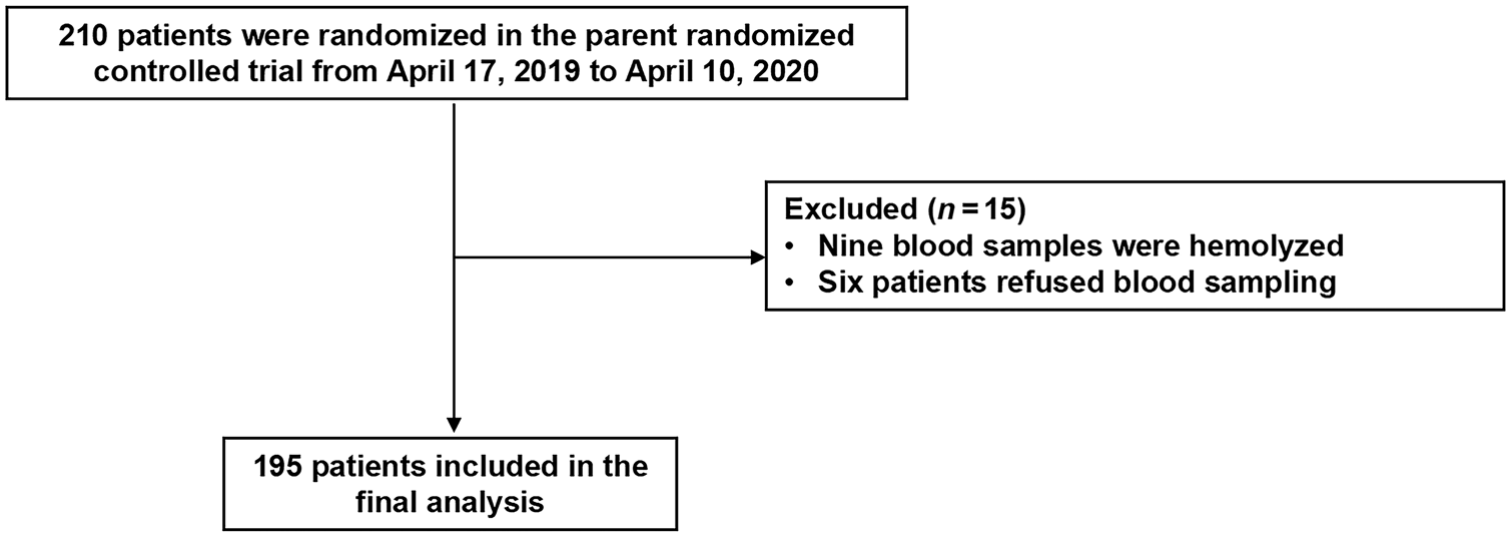
Study flowchart.

Of the 210 participants enrolled in the parent study, 15 were excluded from the final analysis (9 due to hemolyzed blood samples and 6 who declined blood sampling), resulting in 195 patients included in the final analysis (Figure 1). Among these 195 patients, 125 (64.1%) completed delirium assessments for all seven postoperative days, while the remaining 70 (35.9%) were discharged from the hospital before postoperative Day 7.

Patients' characteristics are presented in Table 1. Of this cohort, the mean age was 70.2 ± 4.2 years, 134 (68.7%) were male, and the median duration of education was 9.0 (interquartile range, 5.5, 12.0) years. A total of 26 patients (13.3%) experienced postoperative delirium.

**TABLE 1:** Baseline characteristics and perioperative variables of the study population.

Variables	Total (*n* = 195)
Age, years	70.2 ± 4.2
Male, *n* (%)	134 (68.7)
BMI, kg/m^2^	23.1 ± 2.9
ASA class, *n* (%)	
II	170 (87.2)
III	25 (12.8)
Charlson score	2.0 (2.0, 3.0)
Baseline MMSE score	27.0 (25.0, 29.0)
Duration of education, years	9.0 (5.5, 12.0)
Surgery site, *n* (%)	
Hepatobiliary and pancreatic, *n* (%)	98 (50.3)
Urologic	70 (35.9)
Gynecological	7 (3.6)
Gastrointestinal	20 (10.3)
Duration of surgery, min	175.0 (130.0, 235.0)
Duration of anesthesia, min	216.0 (170.0, 275.0)
Intraoperative consumption of sufentanil, ng/kg/min	2.4 (1.8, 3.3)
Intraoperative consumption of remifentanil, µg/kg/min	0.13 (0.11, 0.14)
Blood loss during surgery, milliliter	100.0 (50.0, 300.0)
With delirum, *n* (%)	26 (13.3)
Highest MDAS	6.0 (4.0, 8.0)

Abbreviations: ASA, American Society of Anesthesiologists; Aβ, amyloid‐beta; BMI, body mass index; MDAS, Memorial Delirium Assessment Scale; MMSE, Minimum Mental State Examination.

### 3.2 The Difference in Aβ42 Levels and Other Cytokines Between Patients With and Without Delirium

**FIGURE 2:**
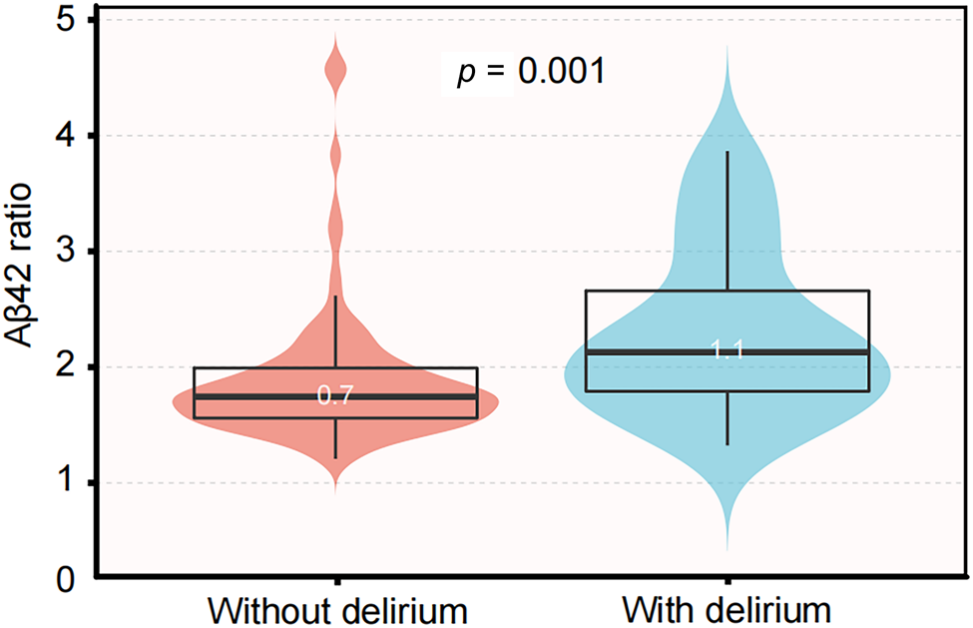
Comparison of the Aβ42 ratio among delirium and non‐delirium groups.

The preoperative plasma Aβ42 level was 11.3 (8.5, 14.6) pg/mL, while the postoperative plasma Aβ42 level was 8.7 (6.3, 12.2) pg/mL. Baseline plasma Aβ42 levels did not differ significantly between patients with or without delirium [9.7 (8.7, 13.1) pg/mL vs. 11.4 (8.4, 14.8) pg/mL, *p *= 0.167]. However, postoperative plasma Aβ42 levels were significantly higher in patients who experienced delirium [10.9 (8.9, 14.6) pg/mL vs. 8.2 (6.2, 11.9) pg/mL, *p *= 0.012], and the Aβ42 ratio (postoperative/preoperative) was significantly elevated in patients with delirium [1.1 (0.8, 1.7) vs. 0.7 (0.6, 1.0), *p* = 0.001] (Figure 2). No significant differences were observed in other preoperative and postoperative plasma cytokines between groups (Table 2).

**TABLE 2:** The comparisons of cytokines between patients with and without delirium.

Variables	Total (*n* = 195)	Without delirium (*n* = 169)	With delirium (*n* = 26)	*p*
Preoperative Aβ42, pg/mL	11.2 (8.5, 14.6)	11.4 (8.4, 14.8)	9.7 (8.7, 13.1)	0.167
Postoperative Aβ42, pg/mL	8.9 (6.3, 12.1)	8.1 (6.2, 11.9)	10.9 (8.9, 14.6)	0.012
Aβ42 ratio	0.8 (0.6, 1.1)	0.7 (0.6, 1.0)	1.1 (0.8, 1.7)	0.001
Preoperative IL6, pg/mL	17.3 (10.6, 39.8)	17.6 (10.7, 42.9)	14.4 (10.1, 26.5)	0.282
Postoperative IL6, pg/mL	47.0 (20.8, 97.1)	46.7 (20.6, 97.2)	57.7 (21.5, 94.4)	0.8
IL6 ratio	1.7 (1.0, 4.6)	1.7 (1.0, 4.2)	1.7 (1.0, 8.3)	0.653
Preoperative IL10, pg/mL	2.4 (1.4, 5.2)	2.4 (1.3, 5.4)	2.4 (1.4, 3.8)	0.53
Postoperative IL10, pg/mL	13.1 (5.9, 24.3)	13.3 (6.1, 24.3)	8.7 (5.5, 19.4)	0.546
IL10 ratio	4.0 (1.8, 11.1)	4.0 (1.8, 10.8)	3.8 (2.1, 13.2)	0.932
Preoperative TNFα, pg/mL	59.2 (35.2, 115.0)	56.2 (34.9, 113.5)	71.7 (40.6, 113.9)	0.681
Postoperative TNFα, pg/mL	61.0 (37.3, 118.6)	57.3 (37.2, 116.9)	66.9 (41.9, 124.7)	0.892
TNFa ratio	1.0 (0.8, 1.2)	1.0 (0.8, 1.2)	0.9 (0.8, 1.1)	0.372
Preoperative NGF, pg/mL	55.7 (32.8, 92.7)	54.3 (33.1, 96.0)	58.3 (33.3, 77.6)	0.902
Postoperative NGF, pg/mL	54.6 (36.7, 92.1)	54.4 (36.7, 96.9)	56.4 (37.7, 86.5)	0.777
NGF ratio	1.0 (0.8, 1.2)	1.0 (0.8, 1.2)	1.0 (0.8, 1.1)	0.621
Preoperative S100β, pg/mL	45.4 (32.6, 77.3)	43.5 (32.5, 76.4)	53.6 (41.1, 79.7)	0.201
Postoperative S100β, pg/mL	87.6 (54.5, 172.0)	81.6 (54.2, 168.9)	123.9 (73.2, 186.7)	0.318
S100β ratio	1.7 (1.1, 3.1)	1.6 (1.1, 3.1)	2.1 (1.2, 3.5)	0.741
Preoperative AQP4, U/mL	10.2 (7.4, 15.6)	10.4 (7.5, 15.6)	9.3 (6.1, 14.7)	0.265
Postoperative AQP4, U/mL	10.9 (7.7, 15.6)	11.1 (7.7, 16.2)	9.2 (7.5, 14.2)	0.289
AQP4 ratio	1.0 (0.8, 1.2)	1.0 (0.8, 1.3)	1.0 (0.8, 1.1)	0.383
Preoperative MMP9, ng/mL	169.8 (114.6, 273.9)	169.9 (116.5, 268.4)	134.8 (110.5, 305.1)	0.744
Postoperative MMP9, ng/mL	190.5 (109.2, 314.5)	179.7 (108.5, 302.2)	241.3 (121.8, 369.5)	0.459
MMP9 ratio	1.1 (0.7, 1.7)	1.1 (0.7, 1.7)	1.0 (0.8, 2.2)	0.59
Preoperative Tau, pg/mL	3.2 (2.0, 7.1)	3.5 (2.1, 7.3)	2.1 (1.7, 3.9)	0.023
Postoperative Tau, pg/mL	3.2 (2.0, 6.9)	3.3 (2.1, 7.4)	2.2 (1.6, 4.2)	0.013
Tau ratio	1.0 (0.8, 1.2)	1.0 (0.8, 1.2)	0.9 (0.8, 1.1)	0.539

*Note*: The cytokine ratios were calculated as the ratio of postoperative to preoperative levels.

Abbreviations: AQP4, aquaporin 4; Aβ, Amyloid‐Beta; IL, interleukin; MMP9, matrix metalloproteinase‐9; NGF, nerve growth factor; TNFα, tumor necrosis factor α.

### 3.3 Association of Aβ42 Ratio With Other Cytokines

Spearman correlation analysis revealed that the MMP9 ratio exhibited a positive correlation with the Aβ42 ratio (ρ = 0.26, *p *< 0.001), and this correlation remained significant after Bonferroni correction (*p *< 0.006; Table S1). No significant correlation was observed between the Aβ42 ratio and the ratios of other cytokines, including IL‐6, IL‐10, TNFα, NGF, S100β, AQP4, and Tau.

### 3.4 Association Between Aβ42 Ratio and Delirium Risk

The results of the univariable regression models are summarized in Table 3. The duration of education, duration of anesthesia, and the randomization group were identified as the potential confounders with *p* < 0.05 in the univariable analysis. The MMP9 ratio was considered a covariate in the multivariable regression model to investigate whether the Aβ42 ratio contributed to the pathogenesis of delirium independently of BBB disruption. The duration of anesthesia was excluded during the covariables screening as the changes in the effect size of the exposure were lower than 10% when included or excluded from the multivariable model. Finally, the randomized group, duration of education, and MMP9 ratio were selected as covariates and were adjusted in the multivariable models. The details of the covariate selection are shown in Table S2.

**TABLE 3:** Univariable and multivariable logistic regression analyses of the association between Aβ42 ratio and delirium risk.

Variables	OR	95% CI	*p* value
Univariable			
Age	1.01	0.91–1.11	0.896
Male	0.84	0.35–2.01	0.694
BMI	1.02	0.89–1.17	0.77
ASA class	1.28	0.4–4.08	0.675
Charlson score	0.99	0.83–1.18	0.901
Baseline MMSE score	1	0.85–1.17	0.964
Duration of education	0.9	0.82–0.99	0.025
Surgery site			
Hepatobiliary and pancreatic	1.18	0.52–2.7	0.694
Urologic	1.37	0.59–3.17	0.465
Gynecological	0	0–Inf	0.992
Gastrointestinal	0.28	0.04–2.19	0.226
Duration of surgery	1	1–1.01	0.05
Duration of anesthesia	1	1–1.01	0.044
Intraoperative consumption of sufentanil	0.88	0.63–1.23	0.461
Intraoperative consumption of remifentanil	0.55	0–142319.5	0.925
Blood loss during surgery	1	1–1	0.16
Randomization group	0.4	0.16–0.97	0.042
Preoperative Aβ42	0.48	0.19–1.23	0.126
Postoperative Aβ42	1.33	0.79–2.24	0.29
Multivariable			
Unadjusted	2.12	1.25–3.59	0.005
Adjusted Model 1^a^	2.7	1.48–4.92	0.001
Adjusted Model 2^b^	3.21	1.71–6.05	< 0.001

*Note*: ASA, American Society of Anesthesiologists; Aβ, amyloid‐beta; BMI, body mass index; CI, confidence interval; MMSE, Minimum Mental State Examination; OR, odds ratio.

^a^ Model was adjusted for the matrix metalloproteinase‐9 ratio.

^b^ Model was adjusted for the randomization group, the duration of education, and the matrix metalloproteinase‐9 ratio.

Multivariable logistic regression analyses assessed the relationship between the Aβ42 ratio and delirium risk, with the results summarized in Table 3. Model 1 was adjusted; Model 2 was adjusted for the MMP9 ratio; Model 3 was additionally adjusted for the randomization group and duration of education. An increased plasma Aβ42 ratio was associated with a higher delirium risk (OR 3.21; 95% CI, 1.71–6.05; *p < *0.001) in the fully adjusted model.

**FIGURE 3:**
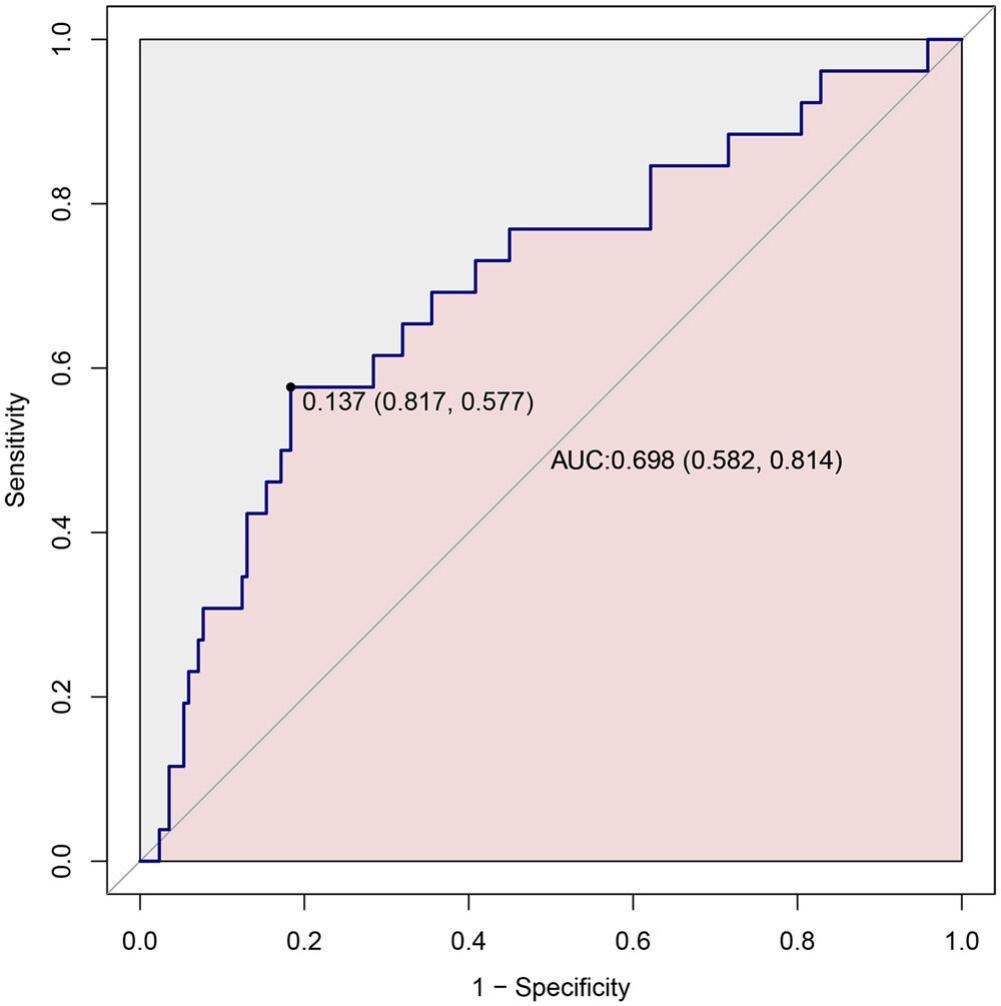
The receiver operating characteristic curve analysis of the Aβ42 ratio for delirium risk. AUC, area under the curve.

The ROC curve analysis demonstrated that the Aβ42 ratio has moderate predictive accuracy for postoperative delirium, with an AUC of 0.698; 95% CI, 0.582–0.814. The optimal cut‐off value was 0.137, with an accuracy of 0.785, sensitivity of 0.577, specificity of 0.817, PPV of 0.326, and NPV of 0.926 (Figure 3).

After the adjusted duration of education and the intervention group in the models, the mediation effect analysis showed that MMP9 mediated only 1.3% of the association between the Aβ42 ratio and delirium risk (indirect effect: *β* = −0.001; 95% CI, −0.014–0.005; *p *= 0.833; direct effect: *β* = 0.089; 95% CI, 0.045–0.135).

### 3.5 Association Between Aβ42 Ratio and Delirium Severity

Table 4 presents the linear regression analysis of the log‐transformed Aβ42 ratio and the postoperative peak MDAS score. Model 1 was adjusted; Model 2 was adjusted for the MMP9 ratio; and Model 3 was additionally adjusted for the randomization group and duration of education. In the fully adjusted model, an increased plasma Aβ42 ratio was associated with more severe delirium (*β* coefficient 3.04; 95% CI, 0.90–5.18; *p *= 0.006).

**TABLE 4:** Multivariable linear regression analyses of the association between the log‐transformed Aβ42 ratio and delirium severity.

Multivariable	*β* (95% CI)	*p* value
Unadjusted	2.45 (0.41–4.5)	0.02
Adjusted Model 1^a^	2.79 (0.56–5.03)	0.015
Adjusted Model 2^b^	3.04 (0.9–5.18)	0.006

Abbreviations: Aβ, amyloid‐beta; CI confidence interval.

^a^ Model was adjusted for the matrix metalloproteinase‐9 ratio.

^b^ Model was adjusted for the randomization group, duration of education, and the matrix metalloproteinase‐9 ratio.

The generalized linear mixed model shows that the Aβ42 ratio could not predict delirium severity changes over time, with a *β* coefficient of 0.39 (standard error: 0.23; 95% CI, −0.06–0.85; *p =* 0.092).

### 3.6 Subgroup Analysis

The subgroup analysis demonstrates the interaction effects among the post hoc specified subgroups, as summarized in Table 5. A significant effect modification was observed in different randomization groups (*p*_interaction_ = 0.005). An association between the Aβ42 ratio and delirium risk was detected in the control subgroup, while in the intervention subgroup (patients randomized into the intervention arm in the parent randomized controlled trial), the association between the Aβ42 ratio and delirium risk was not statistically significant (control subgroup: OR 6.1; 95% CI, 2.3–16.15 and intervention subgroup: OR 1.02; 95% CI, 0.37–2.84).

**TABLE 5:** Subgroup analysis.

Subgroup	Event/total (%)	OR (95% CI)	*p* value	*p*_interaction_
Age				0.343
≤ 70 years	14/110 (12.7)	1.69 (0.83–3.46)	0.15	
> 70 years	12/85 (14.1)	2.84 (1.26–6.43)	0.012	
Group				0.005
Control arm	18/98 (18.4)	6.1 (2.3–16.15)	< 0.001	
Intervention arm	8/97 (8.2)	1.02 (0.37–2.84)	0.967	
Duration of education				0.233
≤ 9 years	21/119 (17.6)	3.24 (1.46–7.18)	0.004	
> 9 years	5/76 (6.6)	1.58 (0.62–4.01)	0.337	

Abbreviations: OR, odds ratio; CI, confidence interval.

### 3.7 Sensitivity Analysis

**FIGURE 4:**
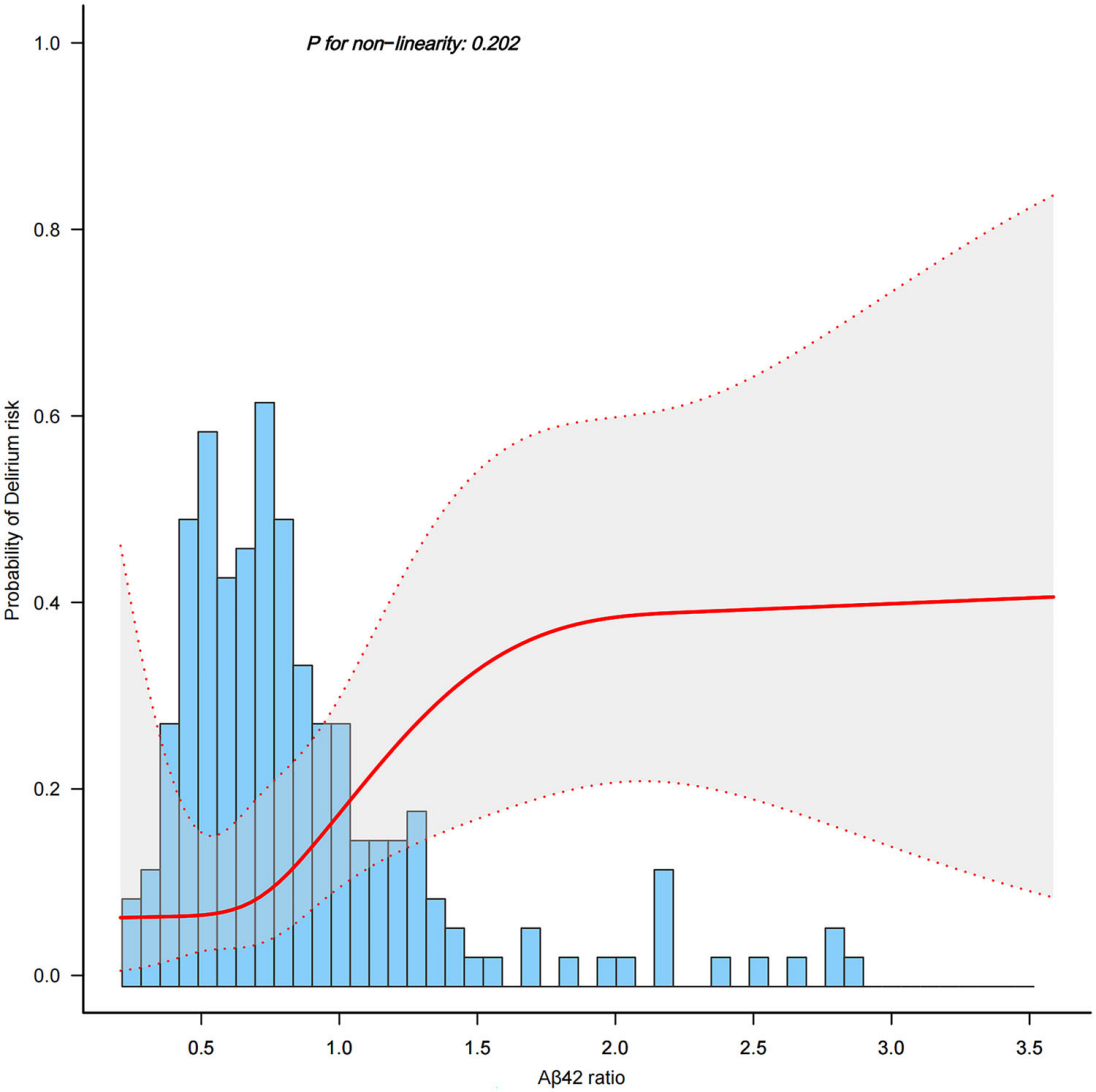
Restricted cubic spline analysis of the relationship between Aβ42 ratio and delirium risk. The solid and dashed lines represent the estimated values and 95% confidence intervals. The model was adjusted with the randomization group, duration of education, and the matrix metalloproteinase‐9 ratio.

The restricted cubic spline shows a linear association between the Aβ42 ratio and delirium incidence after full adjustment (*P*_non‐linearity_ = 0.202, Figure 4). Logistic regression analysis of Aβ42 ratio quartiles with delirium risk showed that patients in the fourth quartile had a higher adjusted OR compared to those in the first quartile (OR 10.08; 95% CI, 2.46–41.4; *p *= 0.001, *p* for trend < 0.001) (See Table S3).

The association between plasma Aβ42 changes and delirium incidence remained significant when considering Aβ42 change as postoperative Aβ42 minus preoperative Aβ42 (see Table S4). Similarly, the association between the Aβ42 ratio and delirium incidence persisted after log‐transforming the Aβ42 ratio (see Table S5).

We calculated an E‐value to assess the impact of potential unmeasured confounding. The estimated *E* value was 5.83, and the lower confidence limit was 2.81, which suggests that our results are robust unless the unmeasured confounder has a higher delirium risk, represented by an OR exceeding 5.83.

## 4 Discussion

This secondary prospective cohort study revealed a linear association between a higher Aβ42 ratio and an elevated risk and severity of delirium in elderly patients undergoing abdominal surgery. The plasma Aβ42 ratio could be a mini‐invasive biomarker for identifying postoperative delirium in elderly patients.

The current study showed no correlation between baseline plasma Aβ42 and the risk of postoperative delirium, consistent with prior research (van den Boogaard et al. 2011). An exploratory observational study with 100 patients in the intensive care unit also documented that preoperative plasma Aβ42 levels were comparable among patients with or without delirium (Payne et al. 2023). This may be attributed to the fact that the baseline concentration of plasma Aβ42 may represent the preoperative neurological status and cognitive function. The present study showed no significant differences in age and preoperative MMSE scores between patients with and without postoperative delirium, indicating a similar baseline cognitive function among the two groups.

Our findings revealed that postoperative plasma Aβ42 levels and Aβ42 ratios were significantly higher in elderly patients who developed delirium compared to those who did not experience delirium. The results regarding plasma Aβ42 changes during the perioperative period were inconsistent in previous studies (Danielson et al. 2021; Payne et al. 2023; Požgain et al. 2022). A cross‐sectional study with 1314 subjects showed that the elevation in plasma Aβ42 levels was more significant in patients with possible cognitive impairment than in those with normal cognition (J. Wang et al. 2018). However, a prospective cohort study with 100 older patients undergoing non‐intracranial surgery concluded no notable alternation in plasma Aβ42 from the baseline to the first postoperative day (Payne et al. 2023). These inconsistencies may be partly due to differences in study cohorts, measurement time points, and testing methodologies. Previous studies indicated plasma Aβ42 levels might increase during the early pre‐pathological phase, preceding amyloid pathology, and subsequently decrease as the disease advances (Botella Lucena et al. 2022; J. Wang et al. 2018).

Our study showed a positive correlation between the plasma Aβ42 ratio and delirium risk. The mechanism underlying the relationship between higher plasma Aβ42 ratio and increased delirium risk remains unclear. Due to the observation cohort study design, it is insufficient to establish causal relationships between the plasma Aβ42 ratio and delirium risk in this study. However, several lines of evidence suggested that there is a dynamic balance between central and peripheral Aβ42 levels, and plasma Aβ42 increased during the pre‐pathological phase before the progression of robust amyloid pathology, and these elevations correlated with growing concentrations of Aβ42 in the CSF (Botella Lucena et al. 2022; Nakamura et al. 2018). The elevated CSF Aβ42 can exacerbate Aβ42 aggravation in the brain and promote neuronal and synaptic receptor dysfunctions (Finch et al. 2020). Higher plasma Aβ42 levels have been linked to lacunar infarcts and the severity of white matter lesions (van Dijk et al. 2004). Furthermore, the plasma Aβ42 levels exhibited a moderate correlation with Aβ‐PET burden and concentrations of Aβ42 in CSF (Nakamura et al. 2018). As plasma Aβ42 is easily detected, using the plasma Aβ42 ratio as a marker for brain Aβ42 deposition, our data may provide evidence for identifying delirium risk and facilitating the diagnosis of delirium. Our immediate postoperative sampling captures these acute‐phase biomarker alterations to establish their causal relationship with subsequent delirium. However, as delirium onset typically peaks 1–3 days post‐surgery (Marcantonio 2017), single early measurements may not fully reflect the biomarker trajectories modulating delirium risk. These findings should be viewed as preliminary; further study is needed to determine whether serial Aβ42 measurements during the postoperative phase can enhance the identification of postoperative delirium risk in elderly patients.

Previous studies suggested that BBB disruption may occur in the brain of patients with delirium (Taylor et al. 2022), potentially facilitating the transfer of Aβ42 from peripheral blood to CSF. However, the mediation effect analysis conducted in this study does not support the hypothesis that MMP9‐mediated BBB disruption serves as a pathway linking Aβ42 to delirium in this population. Other mechanisms, such as direct neurotoxic effects of Aβ42 (Bu et al. 2018) or neurovascular dysfunction (Liu et al. 2025), which compromise the brain's ability to effectively manage Aβ42, may be relevant and need further investigation. Notably, it is appealing that the relationship between the Aβ42 ratio and delirium risk was more robust in the control arm than in the intervention arm, which indicates that acupuncture intervention may exert its anti‐cognitive decline effect by ameliorating Aβ deposition (Liang et al. 2021; Zheng et al. 2021). Future large‐scale prospective studies are required to verify this.

Our study has the following limitations. First, the analysis was derived from a cohort with a limited sample size and was hypothesis‐generating. Second, the ELISA method was used to measure the concentrations of plasma Aβ42. Novel ultra‐precise technologies, such as a single‐molecule array, ultra‐sensitive immune‐magnetic reduction, and immunoprecipitation–mass spectrometry, may perform better and need further validation (X. Wang et al. 2020). Third, CSF specimens were not obtained alongside the plasma Aβ42 analysis. Fourth, the subgroup analysis was post hoc, with a relatively small number of events in the subgroups. Thus, the findings obtained from this analysis should be considered exploratory. Fifth, delirium was assessed once daily in this study. Reports from family members and medical records were also considered during delirium diagnosis. Sixth, unmeasured confounders may still exist in the observational study. However, the high *E* value in this study supports the robustness of the primary findings. Furthermore, plasma Aβ42 was measured only once immediately after surgery. Future studies should include serial measurements to determine whether Aβ42 elevations in patients with delirium are transient and whether their persistence correlates with long‐term outcomes.

## 5 Conclusions

This secondary analysis suggested that a higher plasma Aβ42 ratio was related to an increased delirium risk among elderly patients who underwent major abdominal surgery. Further study is required to determine whether serial plasma Aβ42 measurements during the postoperative period can improve the identification of postoperative delirium risk in this patient population.

## Author Contributions

**Qianqian Fan**: conceptualization, data curation, writing–original draft, writing–review and editing, investigation, visualization. **Yonghui Wang**: data curation, writing–original draft, writing–review and editing, investigation. **Zhihong Lu**: conceptualization, writing–review and editing. **Lini Wang**: data curation, writing–review and editing, investigation. **Xue Yang**: data curation, writing–review and editing, methodology, investigation, validation. **Ziyu Zheng**: conceptualization, formal analysis, writing–review and editing, supervision, software. **Hailong Dong**: conceptualization, writing–review and editing, supervision. **Lize Xiong**: conceptualization, writing–review and editing, supervision. **Chong Lei**: conceptualization, writing–review and editing, writing–original draft, supervision, funding acquisition, project administration, resources.

## Ethics Statement

This study was approved by the Institutional Review Board of Xijing Hospital (No. KY20182080‐F‐1) and registered on ClinicalTrials.gov as NCT03726073. We performed this study according to the ethical principles for medical research involving human subjects detailed in the Declaration of Helsinki. The patients provided their written informed consent to participate in this study.

## Conflicts of Interest

The authors declare no conflicts of interest.

### Peer Review

The peer review history for this article is available at https://publons.com/publon/10.1002/brb3.70501

## Supporting information



Supporting Information

## Data Availability

The datasets used and/or analyzed during the current study are available from the corresponding author upon reasonable request.
