# Supplementary material for: Association Between Plasma Amyloid‐Beta 42 Ratio and Postoperative Delirium in Elderly Patients Undergoing Major Abdominal Surgery: Secondary Analysis of a Randomized Controlled Trial
Source: Brain Behav. 2025 Apr 21;15(4):e70501. doi: 10.1002/brb3.70501 (PMC12012251; doi:10.1002/brb3.70501)
Supplement: Supplementary file 1 — Supporting Information [file BRB3-15-e70501-s001.docx]

**Supplementary Materials**

**Supplementary Tables**

**Supplementary** **Table 1** Correlation of Aβ42 ratio with other cytokine ratios.

| Cytokine | Aβ42 ratio | *p* value |
| --- | --- | --- |
| IL-6 ratio | 0.13 | 0.085 |
| IL-10 ratio | -0.06 | 0.386 |
| TNFα ratio | 0.13 | 0.086 |
| NGF ratio | 0.10 | 0.188 |
| S100β ratio | 0.14 | 0.054 |
| AQP4 ratio | -0.07 | 0.338 |
| MMP9 ratio | 0.26 | <0.001 |
| Tau ratio | 0.02 | 0.777 |

Note: *Aβ* Amyloid-Beta, *IL* interleukin, *TNFα* tumor necrosis factor α, *NGF* nerve growth factor, *AQP4* aquaporin 4, *MMP9* matrix metalloproteinase-9. Spearman’s rank correlation rho and *P*-values are displayed for all cytokines. The cytokine ratios were calculated by dividing the cytokine levels measured postoperatively by those measured preoperatively.

**Supplementary Table 2** Effect size changes and collinearity assessment

| Covariate | Coefficient (forward selection) | Δ% (vs. unadjusted) | Coefficient (backward elimination) | Δ% (vs. full-adjusted) | VIF | Colinearity | Selected as covariate |
| --- | --- | --- | --- | --- | --- | --- | --- |
| Reference | 0.75 | - | 1.21 | - | 1.103 | No | - |
| Duration of education | 0.87 | 15.4% | 1.07 | -11.6% | 1.108 | No | Yes |
| Duration of anesthesia | 0.76 | 1.3% | 1.17 | -3.2% | 1.058 | No | No |
| Randomization group | 0.84 | 11.7% | 1.18 | -1.8% | 1.041 | No | Yes |
| MMP9 ratio | 0.99 | 32% | 0.95 | -21.1% | 1.047 | No | Yes |

Notes: Forward selection: each covariate was individually added to the unadjusted model (baseline coefficient = 0.75); Backward elimination: each covariate was individually removed from the full multivariable model (baseline coefficient = 1.21); Δ% (percentage change): calculated as [(new coefficient - baseline coefficient)/baseline coefficient] × 100%; VIF: variance inflation factor, values < 2 indicate no multicollinearity; MMP9: matrix metalloproteinase-9; Covariate selection criteria: variables were retained if they altered the Amyloid-beta 42 ratio coefficient by >10% or demonstrated clinical relevance.

**Supplementary Table 3** Modified logistic regression analysis of Aβ42 ratio quartiles with delirium risk

| Aβ42 ratio | Case/Total (%) | Unadjusted model | | Adjusted model ^a^ | |
| --- | --- | --- | --- | --- | --- |
|  |  | OR (95% CI) | *p* value | OR (95% CI) | *p* value |
| 0.21-0.58 | 3/49 (6.1) | 1 (Reference) |  | 1 (Reference) |  |
| 0.58-0.76 | 3/48 (6.2) | 1.02 (0.20–5.33) | 0.979 | 1.41 (0.26–7.65) | 0.693 |
| 0.77-1.05 | 5/49 (10.2) | 1.74 (0.39–7.73) | 0.465 | 2.77 (0.85–13.3) | 0.203 |
| 1.06-3.59 | 15/49 (30.6) | 6.76 (1.81–25.23) | 0.004 | 10.08 (2.46–41.40) | 0.001 |
| *p* for trend |  |  | 0.001 |  | <0.001 |

Note: *OR* odds ratio, *CI* Confidence interval. ^a^Model was adjusted for the randomization group, duration of education, and the matrix metalloproteinase-9.

**Supplementary Table 4** Multivariable logistic regression analysis of the association between plasma Aβ42 changes and delirium risk. The plasma Aβ42 changes Aβ42 change were considered as postoperative plasma Aβ42 levels minus preoperative plasma Aβ42 levels.

| Variables | OR | 95% CI | *p* value |
| --- | --- | --- | --- |
| Unadjusted | 1.09 | 1.02–1.16 | 0.010 |
| Adjusted Model 1^a^ | 1.12 | 1.04–1.21 | 0.004 |
| Adjusted Model 2^b^ | 1.13 | 1.05–1.22 | 0.002 |

Note: *OR* odds ratio, *CI* Confidence interval. ^a^ Model was adjusted for the matrix metalloproteinase-9. ^b^ Model was adjusted for the randomization group, duration of education, and the matrix metalloproteinase-9.

**Supplementary Table 5** Multivariable logistic regression analysis of the association between log-transformed Aβ42 ratios and delirium risk

| Variables | OR | 95% CI | *p* value |
| --- | --- | --- | --- |
| Unadjusted | 17.01 | 2.96–97.71 | 0.001 |
| Adjusted Model 1^a^ | 28.93 | 4.36–191.94 | <0.001 |
| Adjusted Model 2^b^ | 46.31 | 6.20–345.89 | <0.001 |

Note: *OR* odds ratio, *CI* Confidence interval. ^a^ Model was adjusted for the matrix metalloproteinase-9. ^b^ Model was adjusted for the randomization group, duration of education, and the matrix metalloproteinase-9.
